# Nanoscale Investigation of Generation 1 PAMAM Dendrimers Interaction with a Protein Nanopore

**DOI:** 10.1038/s41598-017-06435-1

**Published:** 2017-07-21

**Authors:** Alina Asandei, Andrei Ciuca, Aurelia Apetrei, Irina Schiopu, Loredana Mereuta, Chang Ho Seo, Yoonkyung Park, Tudor Luchian

**Affiliations:** 1 Interdisciplinary Research Department, Alexandru I. Cuza University, Iasi, Romania; 2 Department of Physics, Alexandru I. Cuza University, Iasi, Romania; 3 Department of Bioinformatics, Kongju National University, Kongju, South Korea; 4 Department of Department of Biomedical Science and Research Center for Proteinaceous Materials (RCPM), Chosun University, Gwangju, Korea

## Abstract

Herein, we describe at uni-molecular level the interactions between poly(amidoamine) (PAMAM) dendrimers of generation 1 and the α-hemolysin protein nanopore, at acidic and neutral pH, and ionic strengths of 0.5 M and 1 M KCl, via single-molecule electrical recordings. The results indicate that kinetics of dendrimer-α-hemolysin reversible interactions is faster at neutral as compared to acidic pH, and we propose as a putative explanation the fine interplay among conformational and rigidity changes on the dendrimer structure, and the ionization state of the dendrimer and the α-hemolysin. From the analysis of the dendrimer’s residence time inside the nanopore, we posit that the pH- and salt-dependent, long-range electrostatic interactions experienced by the dendrimer inside the ion-selective α-hemolysin, induce a non-Stokesian diffusive behavior of the analyte inside the nanopore. We also show that the ability of dendrimer molecules to adapt their structure to nanoscopic spaces, and control the flow of matter through the α-hemolysin nanopore, depends non-trivially on the pH- and salt-induced conformational changes of the dendrimer.

## Introduction

Dendrimers are polymers distinguished by the presence of regular branching units interacting covalently with a central core, displaying a solvent-filled inner core and a homogenous and multivalent, functional-groups containing, exterior surface, which can be engineered for specific needs^1^. Topologically, the branching units are arranged in concentric-like layers, whose number, usually called generation, increase with each iterative reaction involved in the dendrimer synthesis. As early as 1984, poly(amidoamine) (PAMAM) dendrimers obtained from an initiation core of ethylenediamine were the first complete dendrimer family (generations G = 0–7) to be synthesized and characterized, followed by commercialization in 1990^2^. They are among the most widely studied dendritic structures^3^. For the different generations of PAMAM dendrimers and when viewed in water, the volume of a single dendrimer increases cubically with generation, whereas its mass increases exponentially and the surface groups grow exponentially at each generation^1, 4^.

As a result of their excellent water solubility, reduced toxicity, facile functionalization and topologic tunability, dendrimers are in the forefront of biomedical applications such as gene transfection^5^, drug delivery^6–9^, they are used as imaging agents^10, 11^, nano-drugs^12, 13^, and in chemical sensing^4, 14^.

Dendrimers possess remarkable structural flexibility, which is advantageous when seeking to achieve reproducible and predictable responsiveness to chemical, biological, or physical stimuli. PAMAM dendrimers have internal tertiary amines and external primary amines with pK’a values in ranges of 3–6 and 7–9, respectively^15^, so their biophysical properties are expected to change significantly with pH. The pH-dependent conformational behavior of dendrimers is a subject of debate, however. On one hand, and as proven by SAXS^16^ and SANS^17^ experiments, the radius of gyration of various generations of the PAMAM dendrimers was found pH dependent, in accord with acidic pH-induced repulsion between dendritic branches or dendrimer back-folding at pH > 9, respectively^4, 18^. These findings were also supported by coarse-grained^19, 20^ and atomistic simulations^18, 21, 22^.

On the other hand, other SANS experiments on generations 8 and 4 PAMAM dendrimer^23, 24^ showed only a modest increase in the radius of gyration from pH 10.1 to 4.7, concluding that the radius of gyration (R_g_) is mostly independent of pH, and this again was backed by other theoretical studies^25^.

To extend the realm of dendrimer’s medical and nanobiotechnology applications, new generations of bio-inspired, smart molecular vehicles for the controlled delivery of dendrimers-conjugated biomolecules are needed, and nanopores have emerged lately as ideal candidates.

Nanopores, either solid-state or protein-based, have been proven tremendously useful for the detection, identification, quantification, and characterization of single molecules^26–39^ and in mediating transport of various molecules such as therapeutic agents into cells^40–43^.

In pioneering studies, authors aimed at describing experimentally and from the perspective of molecular modeling, the molecular view of PAMAM (generations G1 to G5) dendrimers under confinement in the α-hemolysin (α-HL) nanopore^44, 45^. To date however, physical aspects of dendrimers confinement into nanoscale domains need further elucidation. Still an open issue, the pH dependence of dendrimer size, conformation and mobility inside nanoscale volumes, as opposed to the bulk solution (*vide supra*), is key for the utilization of PAMAM dendrimers as biomolecule delivery vehicles in physiological environments with nanopores.

Herein we employed electrical recordings across a single α-HL nanopore isolated in a lipid membrane, to provide a deeper insight into the conformational and diffusive properties of the first generation PAMAM dendrimers in nanoconfined volumes, at acidic and neutral pH values, in ionic strengths of 0.5 and 1 M KCl. At a given ionic strength, the reversible dendrimers-α-HL interactions, quantified through the drop in ionic current they cause during translocation through the nanopore, and the average capture and sojourn times inside the nanopore, were found to be voltage- and pH-dependent. The ionic current blockade and kinetic analysis of dendrimer passage through the nanopore demonstrate the existence of a more compacted dendrimer at neutral than at acidic pH. The experimentally derived diffusion constant of the dendrimer inside the α-HL, was found lower at neutral pH (the case of a compacted dendrimer, with a lower radius), as compared to acidic pH (the case of an expanded dendrimer, with a larger radius). Modelling to a first approximation dendrimers as ideal, homogeneous spheres, this is in apparent contrast to predictions derived from the Stokes–Einstein relation, and we further discuss this phenomenon.

The dendrimer entry and transport through the nanopore, as well as the ion current blockade, differ in experiments undertaken at distinct salt concentrations (0.5 M and 1 M KCl), and neutral pH. We show that the ionic current blockade by the dendrimer increases at higher salt concentration. This is counterintuitive, given that the effective size of the electrically charged dendrimer is slightly lower in 1 M KCl than 0.5 M KCl, due to a reduced Debye screening length and thereby a more compacted dendrimer in 1 M KCl. We show that the dendrimer association to the α-HL is dominated by a free energy barrier, and in addition, the activation free energy of dendrimer confinement within the nanopore is higher at a lower salt concentration. The increased friction between the dendrimer and the inner walls of the α-HL, as it is the case when the ionic strength is lowered, leads to a reduced diffusion coefficient of the dendrimer inside the nanopore, as expected.

## Results

**Figure 1:**
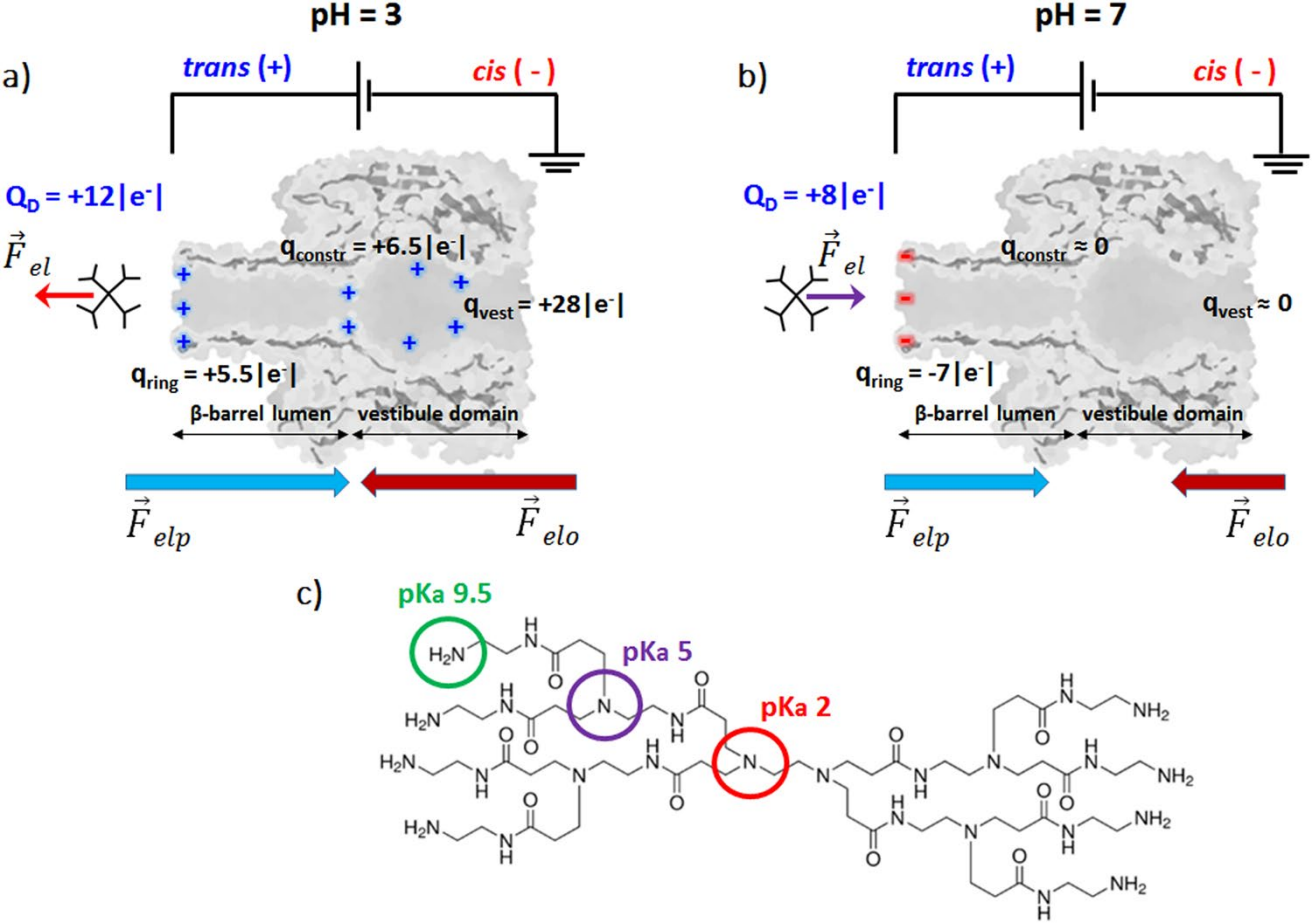
Principle of dendrimer detection using an α-HL nanopore inserted into a lipid membrane. A transmembrane potential difference was applied as shown, with dendrimer on the *trans* side of the membrane, to generate the electrophoretic driving force ($\overrightarrow{{F}_{elp}}$) for dendrimer capture and transport across the nanopore. The sketched interplay of electrophoretic ($\overrightarrow{{F}_{elp}}$) and electro-osmotic forces ($\overrightarrow{{F}_{elo}}$) acting on the PAMAM-G1 dendrimer in acidic (pH = 3; panel a) and neutral pH (pH = 7; panel b) electrolytic solutions are represented. In panel (c) we represented the PAMAM-G1 chemical structure, displaying the eight surface primary amino groups (in green) and six tertiary amino groups (in purple and red), as well as their corresponding pK_a_ values^47^.

In Fig. 1 we represent schematically the structure of the PAMAM-G1 dendrimer used herein, as well as a sketched view of the experimental paradigm for investigating the dendrimers-α-HL interactions.

At pH = 3 (Fig. 1, panel a), the *trans*-added dendrimer (calculated bare charge of Q_D_ = +12|e−|, where |e−| represents the absolute value of the unit electronic charge) interacts with the positive charges at the β-barrel lumen opening (q_ring_ = +5.5|e−|), and inside the α-HL it interacts with the positively charged residues from its constriction (q_constr._ = +6.5|e−|) and vestibule domains (q_vest._ = +28|e−|)^46^. These repulsive electrostatic interactions are represented as $\overrightarrow{{F}_{el}}$, red arrow. At pH = 7 (Fig. 1, panel b), the dendrimer has a calculated bare charge of Q_D_ = +8|e^−^|, and it interacts attractively with the negative electric charge on the β-barrel opening (q_ring_ = −7|e^−^|) ($\overrightarrow{{F}_{el}}$, purple arrow). At this pH, the constriction and vestibule zone are devoid of electrical charge^46^. As shown in Fig. 1, panels a and b, the electrophoretic ($\overrightarrow{{F}_{elp}}$) and electro-osmotic forces ($\overrightarrow{{F}_{elo}}$) act oppositely on the dendrimer, at both pH’s. However, they are reduced in magnitude at neutral as compared to acidic pH, as the charged state of the dendrimer is smaller at pH = 7 than at pH = 3, and the anionic selectivity of the nanopore, which determines the net solvent velocity across the nanopore, is less prevalent in neutral as compared to acidic buffers.

**Figure 2:**
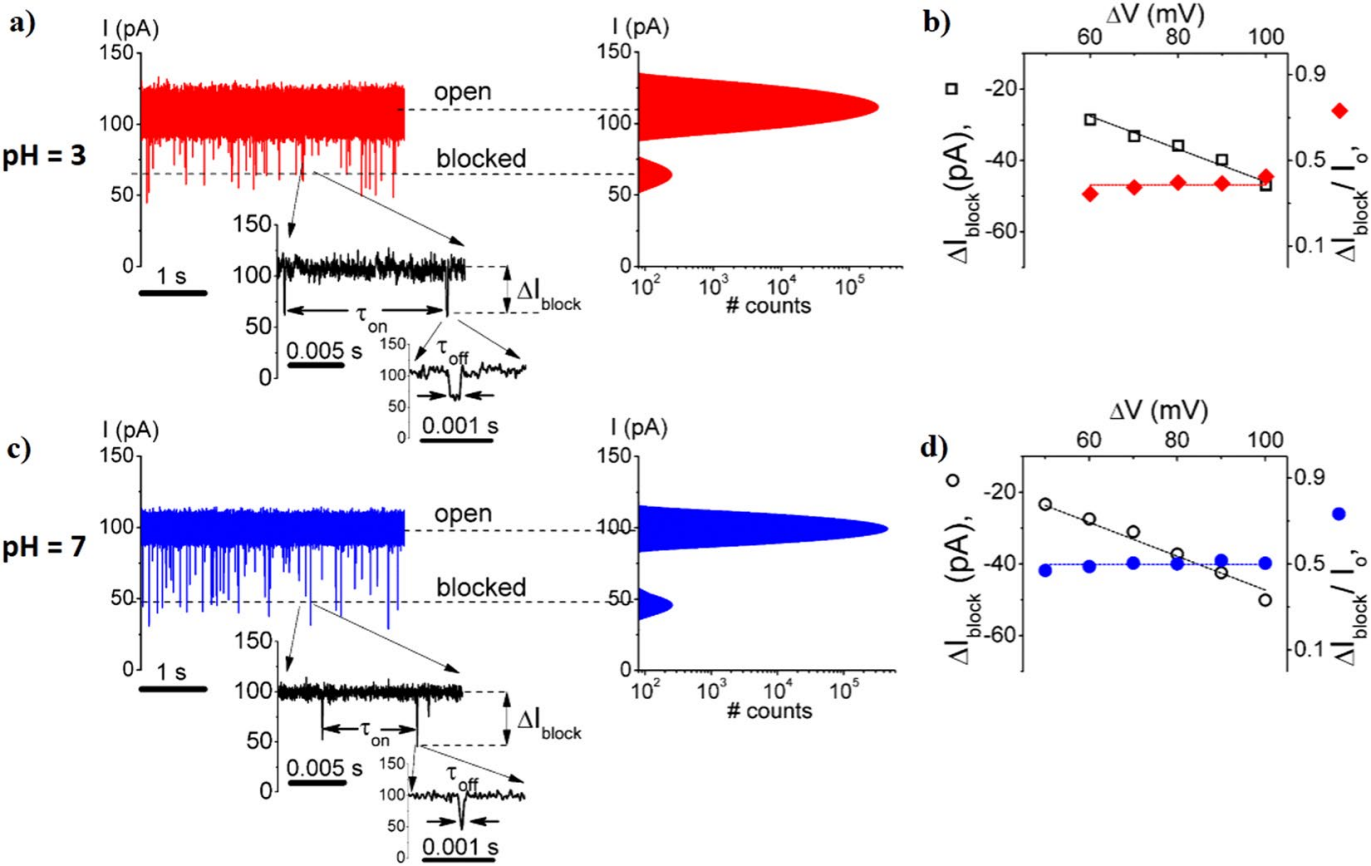
Uni-molecular view of PAMAM-G1 – α-HL interactions at acidic and neutral pH, from electrophysiology traces. Selected ionic current recordings reflecting the PAMAM-G1 – α-HL interactions, and all-events histograms, recorded at ΔV = +100 mV, in 1 M KCl, at acidic (pH = 3; panel a) and neutral pH (pH = 7; panel c). The dendrimer was *trans*-added at a bulk concentration of 500 μM. Indicated are the ionic current levels assigned to the free nanopore (‘open’)(I_o_), and the transiently occupied nanopore by a single dendrimer (‘blocked’)(I_blocked_). The zoomed-in traces illustrate the main parameters used to describe the electrical signature of the PAMAM-G1 – α-HL interactions (*i*.*e*., τ_on_; inter-event time, τ_off_; blockade duration and ΔI_block_; current blockage amplitude). In panels b and d we illustrate the ΔI_block_ vs. ΔV dependence (at pH = 3; empty squares (◽); panel b, and respectively at pH = 7; empty-circles (○); panel d), and that of the relative current blockade (ΔI_block_/I_o_) vs. ΔV (at pH = 3; red-filled diamonds (); panel b, and respectively at pH = 7; blue-filled circles (); panel d).

Single dendrimers interactions with the α-HL generate stochastic reductions of the ionic current across the nanopore, viewed as downwardly oriented current spikes (Fig. 2). The time intervals between successive blockade events (τ_on_), the blockade extent (ΔI_block_) and duration (τ_off_) were statistically analysed within the general theory of Markov processes^48, 49^.

As shown in Fig. 2, the extent of the voltage-dependent, relative current blockade induced by the dendrimer present inside the α-HL was quantified by $\frac{{\rm{\Delta }}{I}_{block}}{{I}_{o}}$, where ΔI_block_ = I_blocked_ − I_o_, and fitted with a straight line of constant (zero) slope, generating a higher value at neutral $(\frac{{\rm{\Delta }}{I}_{block}}{{I}_{o}} = 0.5\pm 0.006; {\rm pH}=7)$ than at acidic pH $(\frac{{\rm{\Delta }}{I}_{block}}{{I}_{o}}=0.39\pm 0.01; {\rm pH}=3)$.

**Figure 3:**
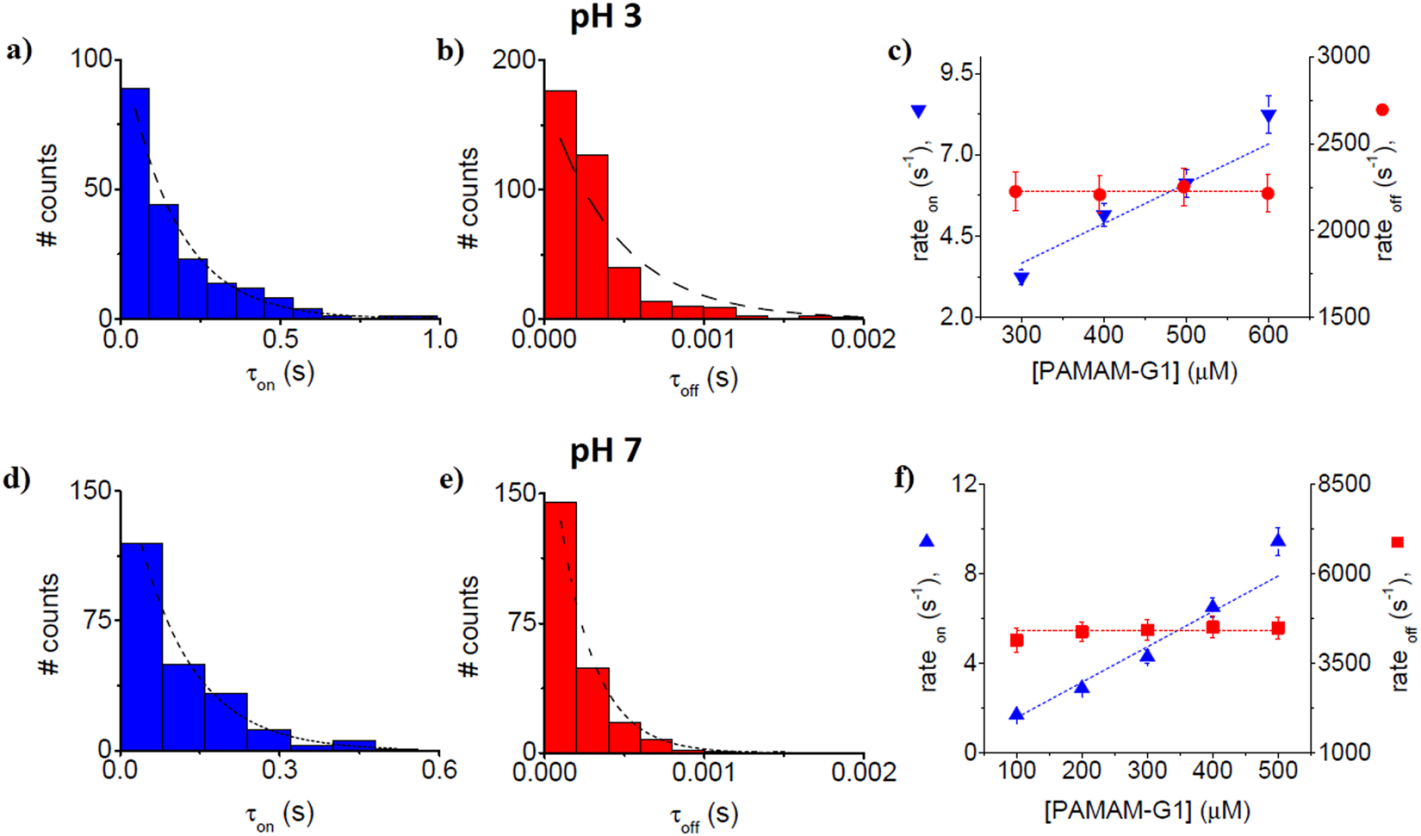
The kinetics description of PAMAM-G1 – α-HL interactions, at acidic and neutral pH. Representative histograms of the inter-event (τ_on_) and blockade duration intervals (τ_off_) characterizing the PAMAM-G1 – α-HL reversible interactions recorded at a transmembrane potential of ΔV = +100 mV, in 1 M KCl, at pH = 3 (panels a and b) and pH = 7 (panels d and e). At distinct concentrations of the dendrimer, such histograms were fitted with a mono-exponential function, and corresponding reaction rates describing the PAMAM-G1 – α-HL interactions were derived.

The dendrimer capture rate by the nanopore (rate_on_) quantified through the inverse value of the average association times (rate_on_ = ${\hat{\tau }}_{on}^{-1}$) and marked by ‘▾’ – ‘down triangles’ at pH = 3 (Fig. 3, panel c) and ‘▴’ – ‘up triangles’ at pH = 7 (Fig. 3, panel f), varied linearly with the increase in the dendrimer concentration. The dissociation rates (rate_off_) measured as the inverse value of the average dissociation times as rate_off_  =  ${\hat{\tau }}_{off}^{-1}$, marked by ‘’ – ‘red circles’ at pH = 3 (Fig. 3, panel c) and ‘’ – ‘red squares’ at pH = 7 (Fig. 3, panel f), remained invariant with the increase in the dendrimer concentration. These observations were suggestive of a bimolecular model of PAMAM-G1 – α-HL interactions, for which the corresponding rates constants are related to the reaction rates using the expressions: *k*_*on*_ = *rate*_*on*_[*PAMAM* − *G*1]^−1^, *k*_*off*_ = *rate*_*off*_. From the linear fit of these data vs. [*PAMAM* − *G*1] with constant (rate_on_), and respectively zero slope functions (rate_off_), we arrived at corresponding values of association and dissociation rate constants (Table 1).

**Table 1:** The association (k_on_) and dissociation (k_off_) rate constants characterizing PAMAM-G1 – α-HL interactions at ΔV = +100 mV, and distinct pH and salt concentrations values used herein.

	pH = 3	pH = 7
1 M KCl	*k*_*on*_ = 12.2 × 10^3^ ± 0.6 × 10^3^ s^−1^ M^−1^	*k*_*on*_ = 15.8 × 10^3^ ± 0.8 × 10^3^ s^−1^ M^−1^
	*k*_*off*_ = 2.2 × 10^3^ ± 9.95 s^−1^	*k*_*off*_ = 4.4 × 10^3^ ± 60.3 s^−1^
0.5 M KCl		*k*_*on*_ = 4.8 × 10^3^ ± 0.4 × 10^3^ s^−1^ M^−1^
		*k*_*off*_ = 2.4 × 10^3^ ± 9.4 s^−1^

**Figure 4:**
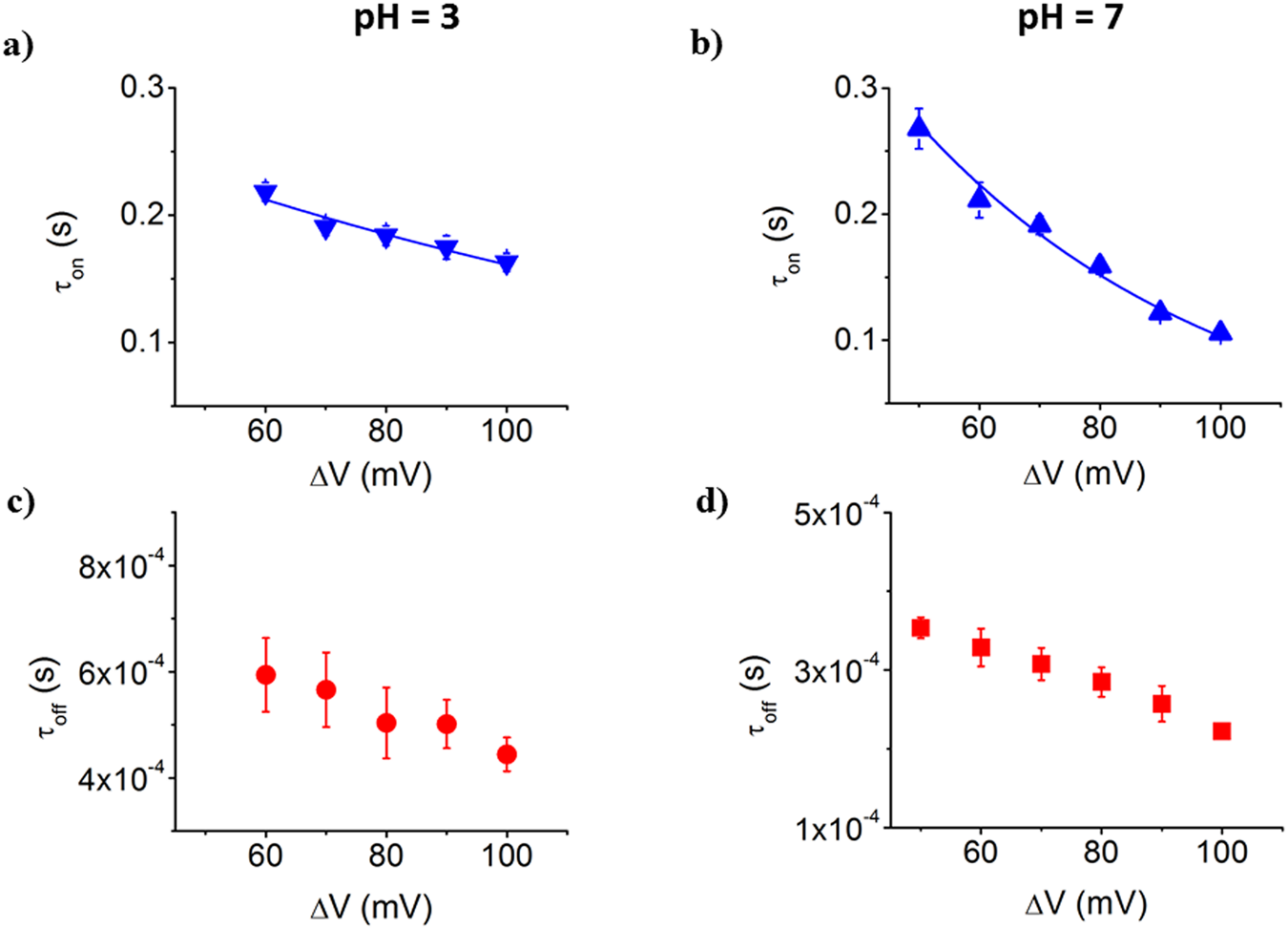
The dynamics of PAMAM-G1 – α-HL interactions, at acidic and neutral pH. The voltage-dependence of inter-event (τ_on_) and blockade duration intervals (τ_off_) characterizing the stochastic ionic current events associated to the dendrimer – α-HL interactions, at a 500 μM concentration of the dendrimer in 1 M KCl, at pH = 3, panels a and c and pH = 7, panels b and d.

The average association and dissociation times of the dendrimer for the nanopore, scales inversely with the applied potential at both pH’s (Fig. 4). The fact that average association times of the dendrimer to the α-HL can be fitted with a van’t Hoff-Arrhenius relationship ${\tau }_{on}=a\cdot \exp (-\,\frac{{\rm{\Delta }}V}{b}),$ demonstrates that the partitioning of the dendrimer from the bulk electrolyte inside the nanopore is an energy barrier-limited process^31^.

Given that the dendrimer dissociates faster from the α-HL at higher applied potentials, leads us to conclude that dendrimer translocates through the α-HL, and this process is approximately twice as fast at pH = 7 than at pH = 3 (Fig. 4, panels c and d). The indirect indication relating analyte translocation by its voltage-dependence dissociation from the nanopore, as used above, was also described and discussed for the case of other analytes^50–55^. Note that for the larger PAMAM-G2 dendrimer, at both pH’s, an increase in the applied potential led to an increase of the dendrimer’s dissociation time (τ_off_) (Fig. S1). In this case, the dissociation process reflects the dendrimer returning to the *trans* side, against the electrophoretic force.

**Figure 5:**
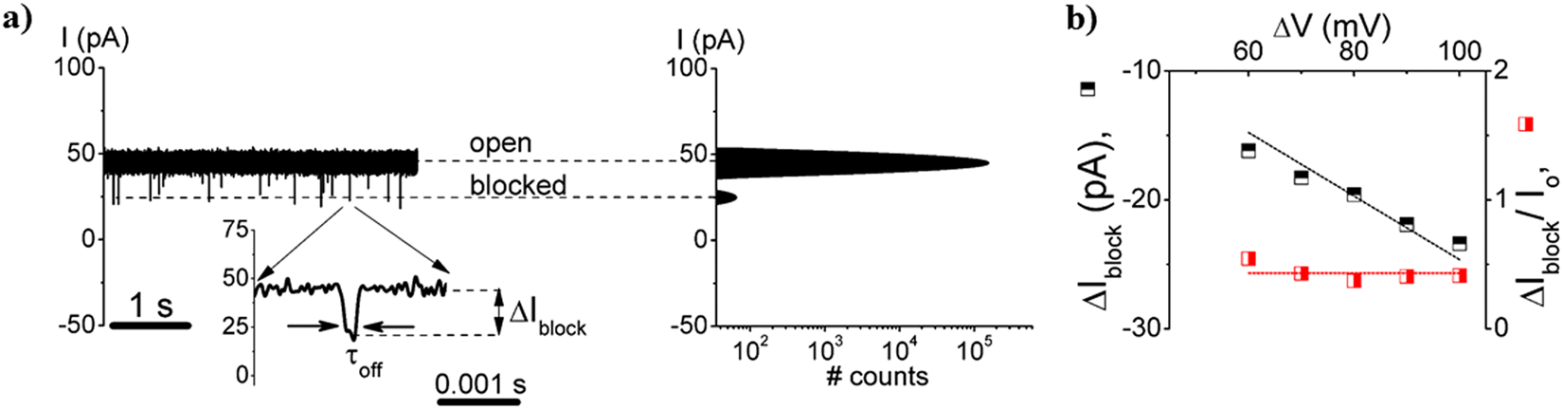
Representative single-channel current trace reflecting the PAMAM-G1 – α-HL interactions in 0.5 M KCl, at pH = 7. In panel a we show a typical electrophysiology trace reflecting the PAMAM-G1 – α-HL reversible blockades recorded at ΔV = +100 mV, in 0.5 M KCl and pH = 7, and the corresponding all-events histogram. The dendrimer (500 μM) was added on the *trans* side of the recording chamber. The zoomed-in trace in the inset depicts the blockade amplitude (ΔI_block_) and the corresponding duration (τ_off_), for a single PAMAM-G1 – α-HL interaction event. In panel b we illustrate the ΔI_block_ vs. ΔV dependence (‘’ – ‘up-filled squares’) and that of the relative current blockade (ΔI_block_/I_o_) vs. ΔV (‘’ – ‘right-filled squares’).

**Figure 6:**
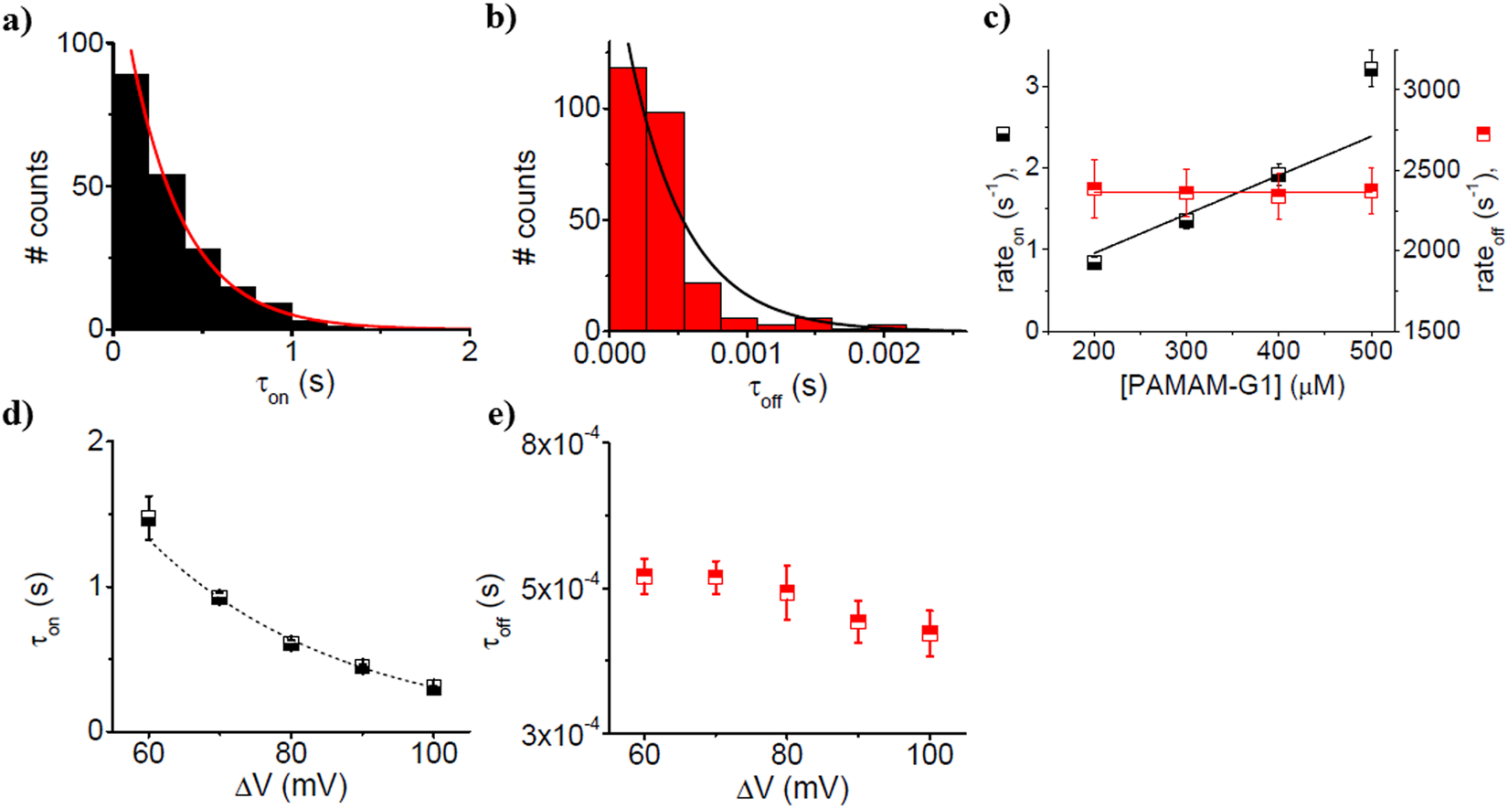
The kinetics description of PAMAM-G1 – α-HL interactions in 0.5 M KCl, at pH = 7. Representative histograms of the inter-event (τ_on_) (panel a) and blockade duration intervals (τ_off_) (panel b) characterizing the PAMAM-G1 – α-HL interactions recorded at ΔV = +100 mV, in 0.5 M KCl, at pH 7, with the *trans*-side added dendrimer (500 μM). The histograms were fitted with mono-exponential functions, and corresponding reaction rates describing the PAMAM-G1 – α-HL interactions were derived. The dendrimer capture rate by the nanopore (rate_on_, ‘’ – ‘down-filled squares’), and the dissociation rate (rate_off_, ‘’ – ‘up-filled squares’) are shown in panel c. The voltage-dependence of inter-event times (τ_on_) and blockade durations (τ_off_) are shown in panels d (‘’ – ‘down-filled squares’) and e (‘’ – ‘up-filled squares’).

To pinpoint the role of electrostatic interactions between the dendrimer and the nanopore, we investigated this process at a lower salt concentration. Typical ionic current traces measured through *α*-hemolysin pore are shown in Fig. 5 and the resulting polymer capture rates are plotted in Fig. 6.

Note that the relative extent of dendrimer-induced blockade of the ionic current $(\frac{{\rm{\Delta }}{I}_{block}}{{I}_{o}})$ vs. ΔV in 0.5 M KCl, was fitted with a zero-slope line of magnitude $\frac{{\rm{\Delta }}{I}_{block}}{{I}_{o}}$ = 0.43 ± 0.03 (Fig. 5), and is 14% lower than that measured in 1 M KCl (Fig. 2).

The dendrimer capture rate by the nanopore (rate_on_) measured in 0.5 M KCl at pH 7 and ΔV = +100 mV (Fig. 6, panel c, ‘’ – ‘down-filled squares’), varies linearly with the increase in the dendrimer concentration, whereas the dissociation rate (rate_off_) (Fig. 6, panel c; ‘’ – ‘up-filled squares’) remains invariant with the increase in the dendrimer concentration, suggestive also of a bimolecular model of PAMAM-G1 – α-HL interactions. As shown above, from the linear fit of these data with constant (rate_on_), and respectively zero slope functions (rate_off_), the corresponding reaction constant rates were derived (Table 1).

The exponential voltage dependency of the dendrimer entry into the nanopore in 0.5 M KCl is consistent with an energy barrier-limited association of the analyte at the nanopore’s β-barrel opening (Fig. 6, panel d; data were fitted with the equation ${\tau }_{on}=a\cdot \exp (-\frac{{\rm{\Delta }}V}{b})$, and the shorter residence time for larger applied voltages (Fig. 6, panel e), is suggestive of dendrimer translocation through the nanopore.

Consistent with the Van’t Hoff-Arrhenius law, the exponential voltage dependence of the dendrimer’s association rate to the mouth of the α-HL’s β-barrel (rate_on_ = ${\hat{\tau }}_{on}^{-1}$) in 1 M (Fig. 4, panel b) and 0.5 M KCl (Fig. 6, panel d), at pH = 7, were fitted according to $rat{e}_{on}=A\cdot \exp (\frac{{Q}_{eff}{\rm{\Delta }}V-{U}^{\ast }}{{k}_{B}{T}_{m}})$ (Fig. S2), where *Q*_*eff*_ denotes the effective electric charge of the dendrimer, U* is the free energy barrier at the entrance of the α-HL pore in the absence of a transmembrane potential, ΔV is the positively biased potential difference across the lipid membrane, k_B_ and T_m_ are the Boltzmann constant and absolute temperature, respectively. The term *A* depends on the dendrimer’s bulk concentration, its diffusion coefficient, and the geometry of the protein pore^31^. During these particular experiments, the dendrimer concentration (500 µM) was similar at both ionic strengths of the electrolytic solution. For a similar value for the dendrimer’s diffusion coefficient in the bulk solution in 0.5 and 1 M ionic strength, one can view the term A as roughly similar in both experimental conditions. Given that rate_on_ can be alternatively written as $rat{e}_{on}={r}_{0}\cdot \exp (\frac{{Q}_{eff}{\rm{\Delta }}V}{{k}_{B}{T}_{m}})$, the term ${r}_{0}=A\cdot \exp (\frac{-{U}^{\ast }}{{k}_{B}{T}_{m}})$ approximates the association rate in the absence of the applied voltage (ΔV → 0), and the effective electric charge of the dendrimer, *Q*_*eff*_, equals *z*_*eff*_|e^−^|, where *z*_*eff*_ is the effective valence of the dendrimer. The resulting values for *r*_*0*_ and *z*_*eff*_, respectively, as determined from the fitted exponential curves in Fig. S2, are summarized in Table 2.

**Table 2:** Estimated values for the capture rate of the dendrimer (r_0_) by the α-HL at equilibrium (ΔV → 0), and the effective valence (quantified by z_eff_) of the dendrimer’s surface in contact with the α-HL’s lumen opening, during capture.

	z_eff_	r_0_ (s^−1^)
1 M KCl (pH = 7)	0.49 ± 0.03	1.40 ± 0.14
0.5 M KCl (pH = 7)	0.94 ± 0.04	0.08 ± 0.01

## Discussion

The ability to control the movement of PAMAM dendrimers in confined nanospaces, by the pH- and salt-dependent charging of the dendrimer, may be of fundamental interest when such analytes are to be used in conjunction with nanopores, as templates for the design of carriers of biomolecules and drugs, novel therapies, toxin inhibitors or biosensors.

### The pH-dependent contributions to the PAMAM-G1-α-HL kinetics

The entry and transport of PAMAM-G1 dendrimers across the α-HL proceeds differently at low and neutral pH values. While the association of PAMAM-G1 dendrimers to the α-HL is described by a Van’t Hoff-Arrhenius law, indicative of an activation barrier for the molecules entry, the association rate constant is slightly higher at neutral than acidic pH. To explain this, we propose that the dendrimer capture at the mouth of the α-HL is governed by at least three factors: (**i**) the electrophoretic force stemming from the transmembrane potential; (**ii**) the electro-osmotic force associated to the net transport of water across the ion-selective α-HL; (**iii**) the electrostatic interaction of the dendrimer with the β-barrel of the α-HL on the *trans*-side and the pH-dependent dendrimer geometric size, which influences the analyte’s subsequent partition into the α-HL’s β-barrel.

As for the electrophoretic component (**i**), we can safely ignore its contribution as a determinant factor for the increase in the association rate constant at neutral, as compared to acidic pH. The reason is that the higher degree of protonation of dendrimer’s amine moieties at pH = 3 as compared to pH = 7, would translate into a larger electrophoretic force acting on the dendrimer at acidic pH, and subsequent augmented association of the analyte to the nanopore as compared to neutral pH.

Given that the α-HL enhances its anion selectivity at pH = 3 as compared to neutral pH (${P}_{{K}^{+}}/{P}_{C{l}^{-}}$ ~ 0.453 at pH = 3 and ${P}_{{K}^{+}}/{P}_{C{l}^{-}}$ ~ 0.703 at pH = 7, see also Fig. S3), an augmentation of the electro-osmotic flow (**ii**) ensues at low pH, and this was proven to influence the analyte partitioning inside the α-HL^49, 56^. For the present case of a *trans* side, positively biased α-HL, the electro-osmotic flow is *cis*-to-*trans* oriented, opposite to the dendrimer motion toward the α-H’s β-barrel. As a result, the lower electro-osmotic flow through the positively voltage biased α-HL, at pH = 7 as compared to pH = 3, may account partly for the slightly larger ability of the dendrimer to associate to the α-HL at neutral pH. The fact that the electro-osmotic flow is a non-negligible contributor to the dynamics of dendrimer capture by the α-HL, is strongly supported by the fact that in a more alkaline buffer (pH = 10.3), which renders the dendrimer almost uncharged and not affected by the electrophoretic force, the dendrimer-α-HL interactions are still visible (Fig. S4). To explain this, we recall that under such alkaline conditions, the α-HL reverses its selectivity and becomes cation selective^57^, and the direction of the electro-osmotic flow reverses as compared to the previous cases explored herein. This in turn promotes the effective capture and transport of the dendrimer through the nanopore at pH = 10.3, despite the fact the electrophoretic force acting on the dendrimer or other electrostatic-related dendrimer-nanopore interactions, become vanishingly small. The non-negligible role played by the electro-osmotic force on setting the analyte dynamics across the nanopore was reported previously^58–63^, and we demonstrated that the electro-osmotic force can capture a peptide at the entry of the α-HL and transiently trap it inside it, against the electrophoretic force^56^.

Electrostatic- and confinement-related contributions (**iii**) can also determine the pH-dependent PAMAM-G1-α-HL interactions. As sketched in Fig. 1, the net charge on the α-HL’s β-barrel opening on the *trans* side, goes from ~−7 |*e* ^−^| at pH = 7 to ~+5.5 |*e* ^−^| at pH = 3, and on the dendrimer from ~+8 |*e* ^−^| at pH = 7 to ~+12 |*e* ^−^| at pH = 3. Consequently, the dendrimer-α-HL electrostatic, attractive interactions could contribute and lower the free energy barrier associated to the dendrimer entry into the nanopore at neutral, as compared to acidic pH. It should be noted however that such interactions manifest themselves only when the two systems (the dendrimer and the α-HL) come at a sub-nanometer distance, since all electrostatic charges are screened by the counterions in the electrolyte, and the Debye screening length κ^−1^ ~ 0.3 nm at 1 M KCl and a temperature of 300 K. Note that even in pure water at a room temperature of T_m_ = 300 K, the relevant scale where the Coulomb energy between two elementary electric charges is balanced by the thermal fluctuation energy, is given by the Bjerrum length (l_B_), which equals ~0.7 nm^64^. On the other hand, as we show in Fig. 2, panels b and d and the discussion below, our data suggest a more compacted dendrimer at neutral than acidic pH, which is also supported by other reports^65^. We therefore posit that a lesser confinement penalty for the dendrimer entry inside the α-H’s β-barrel exists at pH = 7 as compared to pH = 3. In further support, it was suggested that below pH 6, the protonation of tertiary amine groups and the ensuing charge-charge repulsion, renders the PAMAM dendrimer more rigid^66, 67^, which imposes an additional confinement-related penalty for the dendrimer squeeze inside the α-HL’s β-barrel at pH = 3, as compared to a neutral one.

### PAMAM-G1 dendrimers are more compact in neutral than acidic electrolytes

As stated in the ‘*Introduction*’, the pH-dependent size of various generations of the PAMAM dendrimers, is still being disputed. To further shed light on this, we sought to correlate the amplitude of current blockades associated to PAMAM-G1-α-HL interactions, recorded at uni-molecular level and distinct pH values, with the dendrimer size.

Qualitatively speaking, the extent of the current blockade generated by the inclusion of an analyte inside a cylindrical nanopore, ΔI_block_, is directly proportional to the electrolyte volume excluded by the analyte (δ) (${\rm{\Delta }}{I}_{block} \sim \frac{{\sigma }_{buffer}{\rm{\Delta }}V\delta }{{l}_{p}^{2}}$, where σ_buffer_ represents the conductivity of the electrolyte, ΔV is the transmembrane potential across the nanopore, and l_p_ is the nanopore’s length)^68^. The reader should remember however, that the applicability of this formula is not straightforward. One notable difficulty in applying it to faithfully evaluate the analyte volume from the Δ*I*_*block*_, lies in that for a nanopore shape departed from the cylinder geometry, the current blockade varies with the molecule position inside the nanopore, becoming more prevalent as the cross-sectional domains of the nanopore get smaller^69, 70^, and the net charge on a particle can contribute to the current blockade^71–74^.

As shown in Fig. 2, we noted a larger relative blockade ($\frac{{\rm{\Delta }}{I}_{block}}{{I}_{o}}$) at neutral as compared to acidic pH, establishing in the first place that the overall size of the dendrimer changes with pH. Based upon the formula presented above (${\rm{\Delta }}{I}_{block} \sim \frac{{\sigma }_{buffer}{\rm{\Delta }}V\delta }{{l}_{p}^{2}}$) and at a first glance, this experimental observation would suggest the existence of a more expanded dendrimer at neutral (due to the large $\frac{{\rm{\Delta }}{I}_{block}}{{I}_{o}}$) as compared to acidic pH (due to the small $\frac{{\rm{\Delta }}{I}_{block}}{{I}_{o}}$). However, an in-depth analysis done as previously^45^, correlates the data presented in Fig. 2 with the existence in fact of a *more compacted dendrimer at pH* = *7 than pH* = *3*. Knowing that he maximum amount of dendrimer-induced blockade is expected to occur within the ~0.5 nm in length and ~1.5 nm limited aperture of the constriction region the nanopore^75^, a larger relative blockade recorded at pH = 7 than pH = 3 suggests that a more compacted dendrimer exists at pH = 7 than 3, enabling it to more favourably lodge within the constriction region the nanopore at pH = 7 than 3. The presence of the dendrimer within the constriction region the nanopore is also electrostatically favoured at neutral as compared to acidic pH, given the un-charged state of the α-HL’s constriction region on the former, as compared to the latter case (see Fig. 1). The fact that the dendrimer dissociation rate from the nanopore (rate_off_) is larger at neutral than at acidic pH (Fig. 4), is again an indication of the more compacted dendrimer at neutral pH, which helps it squeeze through the constriction region of the α-HL.

### The PAMAM-G1’s diffusion coefficient inside α-HL’s nano-volume scales inversely with its size

To the best of our knowledge, only a few simulation studies have addressed the problem of dendrimer diffusion, and these applied to bulk conditions alone^76–78^. Herein, the residence time of the dendrimer inside the α-HL (τ_off_) was modelled as the first passage time for one-dimensional diffusion of a charged particle in a constant electric field and the electro-osmotic flow through the nanopore^49, 68^. Based on the theoretical formalism and assumptions outlined in the *Supplementary Information* (*‘Uni*-*dimensional formalism for the derivation of the dendrimer drift velocity inside the* α-*HL nanopore*, *under the collective influence of electro*-*osmotic and electrophoretic forces’*), we estimated the values of the dendrimer’s diffusion coefficient inside the nanopore (Table S1). Since the dendrimer-induced current blockades through the α-HL display a linear dependence vs. ΔV, regardless of the working conditions (Figs 2 and 6), we exclude the voltage-induced, partially (un)folded conformations of the dendrimer at distinct applied potentials, so that the viscosity contributions to the diffusion process remain invariant vs. ΔV. In accord with the data presented in Table S1, the average, lower limits of the diffusion coefficient, calculated for all experimental conditions employed, were: D = 3.04E-8 ± 0.27E-8 cm^2^ s^−1^ (1 M KCl, pH = 3), D = 1.78E-8 ± 0.19E-8 cm^2^ s^−1^ (1 M KCl, pH = 7), D = 1.12E-8 ± 0.08E-8 cm^2^ s^−1^ (0.5 M KCl, pH = 7).

At least two important remarks are pertinent at this point: (1) the calculated values of PAMAM-G1 diffusion coefficients inside the α-HL, are roughly two orders of magnitude lower than those estimated for dye-labelled, PAMAM-G1 dendrimers moving in free-standing silica colloidal crystals, containing nanopores with diameters larger than 14 nm^79^ or in bulk solution^80^ (2) quite unexpectedly, despite the herein established dendrimer’s *more compacted geometry at neutral than acidic pH*, the diffusion coefficient of the dendrimer inside the α-HL was estimated to be in fact approximately two-times *larger at pH* = *3 than at pH* = *7*. This is counterintuitive at least in relation to the Stokes–Einstein formula, regarding the dependence of diffusion coefficient D on the analyte radius (${\rm{D}}=\frac{{{\rm{k}}}_{{\rm{B}}}{{\rm{T}}}_{{\rm{m}}}}{6{\rm{\pi }}{\rm{\eta }}r}$, for the diffusion in a medium with viscosity η of a spherical particle of radius r and volume δ_volume_; ${\rm{r}}=\sqrt[3]{\frac{3{{\rm{\delta }}}_{{\rm{volume}}}}{4{\rm{\pi }}}}$ ).

To explain this, we suggest that electrostatic interactions between the charged dendrimer and the inner walls of the nanopore are major determinants of the overall friction experienced by moving dendrimers, aside from hydrodynamic effects contained in the Stokes–Einstein relation. In this scenario, the electrostatic landscape inside the nanopore and electrolyte movement can fine-tune the viscosity-related, friction term entering the Stokes–Einstein relation. As suggested qualitatively in Fig. S5, the electrostatic attractive interactions between the nanopore-captured, *trans*-to-*cis* moving dendrimer, and the nanopore’s β-barrel at neutral, as opposed to acidic pH (when these interactions become overall repulsive), contribute to a smaller diffusion coefficient of the dendrimer under neutral, in comparison to acidic conditions. Previous research has suggested that for flexible and porous polymers (as dendrimers), the average geometrical size is not necessarily a faithful measure of the diffusion coefficient, in the sense that Stokes–Einstein relation may not apply strictly^78^. The effect of electrostatic interactions between the analyte and nanopore on analyte’s translocation was demonstrated in another work, which showed that the single-file DNA transit through functionalized solid-state nanopores is slowed-down at low pH’s, through the acidic pH-induced, increasingly positive charged state of the inner surface of the nanopore^81^.

### The salt effect on the dendrimer geometry. Low salt buffers promote more expanded PAMAM-G1 dendrimers than high salt buffers

We recorded a larger relative blockade $(\frac{{\rm{\Delta }}{I}_{block}}{{I}_{o}})$ induced by the PAMAM-G1 dendrimer in electrolytes containing 1 M KCl (Fig. 2) than 0.5 M KCl (Fig. 5), at pH = 7. As above, we draw attention on the difficulty to apply the formula above to un-equivocally evaluate the analyte’s volume from the ∆I_block_^71–74^.

We note that electrostatic repulsive interactions among the positively charged moieties in dendrimer, determine via the Debye screening, its overall conformation. In high salt electrolytes, when such electrostatic effects are reduced upon counterion condensation, the size of the dendrimer becomes lower than in low salt^23^. It would be therefore expected that the excluded volume of electrolyte by a dendrimer inside the α-HL and ionic current blockade, become correspondingly lower in more concentrated than less concentrated electrolytes. As presented in Figs 2 and 5 however, the opposite is seen. We propose that a candidate mechanisms which may account for the slightly larger degree of current obstruction through the α-HL in 1 M as opposed to 0.5 M KCl, lies in that a less expanded dendrimer in 1 M than 0.5 M KCl is more likely to fully enter the constriction region of the nanopore, and exert the largest extent of ionic current blockade. This finding is similar to previous ones, when the interactions between specific charged peptides and the α-HL pore were studied and evaluated in distinct salt solutions^33^.

### The salt effect on the kinetics of PAMAM-G1-α-HL interactions

In kinetic terms and as shown in Figs 3 and 6, at neutral pH, the 1 M KCl-containing buffer augments the association rate constants of dendrimer-α-HL interaction, as compared to the 0.5 M KCl buffer. From an entropic perspective alone, in low as opposed to high salt buffers, the dendrimer is overall more rigid, partly due to stronger repulsive electrostatic interactions among the positively charged amine moieties, mediated by the lesser Debye screening^66, 67^. Thus, low salt buffers entail an enhanced confinement-related penalty associated to the free energy needed to change the spatial conformation of the dendrimer, to accommodate it inside the constrictive inner volume of the nanopore.

On the other hand, in 0.5 as opposed to 1 M KCl-containing buffer, the attractive electrostatic interactions between the dendrimer and the α – HL, are augmented due to the increase in the Debye length. Note that this is true, since the corresponding Debye lengths in the two conditions are κ^−1^ ≈ 3 Å at 1 M KCl and κ^−1^ ≈ 4 Å at 0.5 M KCl, respectively, which are comparable to difference between the average diameter of the α – HL’s β-barrel (~20 Å) and that of the dendrimer itself (~18 Å). Consequently, and mediated by electrostatic-related effects, this would result in a net increase of the peptide capture rate - via a decrease in the free energy barrier of dendrimer association to the pore - in low, as opposed to high salt containing buffers. Note that this tendency is opposite to the confinement-related contribution presented above.

To conciliate the opposing electrostatic and confinement effects on PAMAM-G1 association to the nanopore, and having established that the dendrimer’s apparent radius is smaller in 1 M than 0.5 M KCl, we propose that in 1 M KCl, less restrictive confinement effects are dominant and lead to the overall augmentation of the dendrimer partitioning into the α-HL’s β-barrel, as compared to 0.5 M KCl. This is also in agreement with previous work, in which the capture and transport of dextran sulfate molecules^82^ or peptide through a α-HL protein^33^, were shown to diminish as the buffer’s ionic strength decreased.

The average value of residence times of the dendrimer inside the α-HL in 0.5 and 1 M KCl, measured at pH = 7 and various ΔV’s, were analysed within the framework of the first passage time for one-dimensional diffusion formalism (*Supplementary Information*), and the lower limit of average values of the diffusion coefficient were obtained (D = 1.78E-8 ± 0.19E-8 cm^2^ s^−1^ (1 M KCl, pH = 7), and D = 1.12E-8 ± 0.08E-8 cm^2^ s^−1^ (0.5 M KCl, pH = 7). The observation that the dendrimer’s diffusion coefficient almost doubles in 1 M as compared to 0.5 M KCl, is attributable at least in part to the reduced friction of the dendrimer inside the nanopore, through more screened electrostatic interactions between the inner walls of the α-HL and the dendrimer, in high as compared to low salt buffers.

### Low-salt, as opposed to high-salt buffers, augment the energy barrier associated to the dendrimer entry inside the α-HL

As presented in Fig. S2, one could model the capture mechanism of dendrimer molecules by the α-HL within the framework of the classical Kramers’ theory, and estimate the association rate constants of the dendrimers to the α-HL’s lumen at pH = 7, in buffers containing 1 M KCl or 0.5 M KCl, near equilibrium conditions (ΔV → 0) (Table 2). The effective valence of the dendrimer is higher in 0.5 as compared to 1 M KCl buffers (Table 2), consistent with the reduced degree of charge screening under low ionic strength buffers. Note that z_eff_ is much lower than the bare charge of the dendrimer molecules, and this may be accounted for by the fact that most of the charges on the dendrimer are not in a region of the β-barrel’s mouth during entry, as suggested in similar cases^83–85^.

By calculating the ratio of *r*_*0*_ values for the two ionic strengths employed (Table 2) $(\frac{{r}_{0}^{1M}}{{r}_{0}^{0.5M}}=\exp (\frac{{U}^{\ast 0.5M}-{U}^{\ast 1M}}{{k}_{B}{T}_{m}}))$, we determined the difference in the energy barrier U* for the two conditions, as ${\rm{\Delta }}{U}^{\ast }={U}^{\ast 0.5M}-{U}^{\ast 1M}={k}_{B}{T}_{m}\,\mathrm{ln}(\frac{{r}_{0}^{1M}}{{r}_{0}^{0.5M}})$. At a room temperature of T_m_ = 295 K, we derived Δ*U* ^*^ = 1.69 kcal mol^−1^, as a quantitative measure for the energy barrier U* difference associated to the dendrimer entry inside the α-HL in a low as compared to a high ionic strength buffer, at equilibrium (ΔV = 0). At this point, it’s worth noting that by measuring the salt-dependence of ssDNA and dsDNA capture by the ClyA nanopore, authors have demonstrated that the capture mechanism (i.e., diffusion-limited for the dsDNA and barrier crossing for the ssDNA, respectively) depends upon the spatial conformation of the analyte, namely a rigid rod for the dsDNA and coil conformation for the ssDNA^73^. Moreover, the fact that decreasing ionic strength entails an increase of the persistence length of DNA, adds up to the complexity of such findings^86^.

In summary, the key findings of our work are threefold: (i) the dynamics of reversible interactions between PAMAM-G1 dendrimers and the α-HL nanopore is sensitive to pH and ionic strength changes in the electrolyte solution, by a fine interplay among conformational and rigidity changes on the dendrimer structure, and the ionization state of the dendrimer and nanopore. We established that low pH values and low salt concentrations in the electrolyte solution, augment the effective size of the dendrimers used herein; (ii) more compact PAMAM-G1 dendrimers, in neutral as compared to acidic buffers, diffuse slower inside the α-HL’s nanoscopic interior, suggestive of the non-Stokesian diffusive behaviour; (iii) the dendrimer-induced blockade on the ionic current through the nanopore, and therefore its ability to occupy nanoscopic spaces, depend non-trivially on the pH- and salt-induced conformational changes of the dendrimer. To conclude, our study highlights the importance of pH- and salt-induced physical changes on the dendrimer structure, which can tailor its self-diffusion and the flow of matter through nanopores. This is especially useful in: (i) instances where such hyperbranched polymers will be at the core of improved therapeutic approaches, as multivalent blockers of matter flow through pore forming exotoxins excreted by particular lethal bacterial strains, or (ii) as nanocarriers in ‘encapsulation and release’ applications, given the important role played by electrostatic interactions in establishing meta-stable analytes-dendrimer complexes^87^.

## Materials and Methods

The PAMAM dendrimers of generation 1 and 2, with an ethylenediamine core (2-carbon core) and 8 surface primary amino groups (PAMAM-G1), and 16 surface primary amino groups, respectively (PAMAM-G2), were manufactured by Dendritech®, Inc. and purchased from Sigma-Aldrich, Germany.

A phospholipid membrane bilayer made from 1,2-diphytanoyl-sn-glycerophosphocholine (Avanti Polar Lipids, Alabaster, AL) dissolved in n-pentane (HPLC-grade, Sigma–Aldrich, Germany) was formed using the Montal-Muller technique^88^ across a ~ 120 μm diameter orifice formed in a 25 μm-thick Teflon film (Goodfellow, Malvern, MA), pretreated with 1:10 hexadecane/pentane (HPLC-grade, Sigma–Aldrich, Germany), separating the *cis*-chamber (grounded) and the *trans*-chamber of the recording cell^36^. The experiments were conducted at room temperature of ~22 °C in 1 M and 0.5 M KCl, buffered with 5 mM MES for pH = 3, 10 mM HEPES for pH = 7 and 10 mM CAPS for pH = 10.3. The self-assembly and inclusion of a single α-HL heptameric nanopore into the phospholipid bilayer was obtained by adding in the *cis*-chamber small volumes (~1 μL) of α-HL protein monomeric solution, from a stock solution made in 0.5 M KCl. After the insertion of a single, stable α-HL pore into the planar lipid membrane, seen as a jump in the ionic current from ~0 to ~100 pA at an applied transmembrane potential ΔV = +100 mV, for 1 M KCl solutions, the PAMAM-G1 dendrimer was added in the *trans*-chamber from a 10 mM stock solution made in methanol, to achieve final concentrations varying from 100 to 600 μM. For the experiments involving the PAMAM-G2 dendrimer, the analyte was added from a stock solution made in methanol (20 mM), at a *trans* concentration of 500 μM. Note that in this study, the particular choice of dendrimer addition side was made as to facilitate conclusions to be drawn within the theoretical frame presented previously^45^. With a reversed polarity of the transmembrane potential, *cis*-added dendrimer interactions with the nanopore are visible as well (not shown here). In other experiments performed with charged peptides^56^, we demonstrated that such small analytes do interact with the nanopore from either side.

Measurements were carried out by applying *trans*-positive voltages ranging between +50 ÷ +100 mV, and the resulting currents reflecting the dendrimer reversible blockades of the α-HL pore were current-to-voltage converted and amplified with an Axopatch 200B (Molecular Devices, USA) instrument, digitized at a sampling frequency of 80 kHz with a NI PCI 6221 16-bit acquisition board (National Instruments, USA), and low-pass filtered at 10 kHz. In order to reduce the effect of environmental noise, the recording system was shielded in a Faraday cage (Warner Instruments, USA), and placed on a vibration-free platform (BenchMate 2210, Warner Instruments, USA). A virtual instrument was developed within LabVIEW 8.20 platform (National Instruments, USA), to facilitate the control and recording of the electrical signals. All numerical analysis and graphic representations of the recorded data were done using pClamp 6.03 (Axon Instruments, USA) and Origin 6 (Origin Lab, USA).

To enable increased accuracy of the analysis of low pass-filtered current blockade events, apparent more frequent at pH = 7 (Fig. S6), the analysis of dissociation times was performed as described previously^89^.

### Determination of α-HL’s ion selectivity

The ion selectivity of the free and dendrimer-blocked α-HL, respectively, was determined from electrophysiology recordings of the ion current mediated by the nanopore as a function of voltage, using a salt gradient of 0.1 M KCl (*cis*)/3 M KCl (*trans*). The Ag–AgCl electrodes were connected to the bilayer chamber via salt bridges made of agarose (~1% w/v) dissolved in 3 M KCl. The electrolyte solutions were buffered with 10 mM HEPES and 5 mM MES for the measurements performed at pH = 7, and at pH = 3, respectively. Current–voltage (I-ΔV) diagrams were used to determine the reversal potential (Ψ_rev_) of the free (${{\rm{\Psi }}}_{rev}^{\alpha -HL}$) and dendrimer-blocked α-HL (${{\rm{\Psi }}}_{rev}^{\alpha -HL+PAMAM-G1}$), respectively, at both pH values (Fig. S3). The charge selectivity of the nanopore (${P}_{{K}^{+}}/{P}_{C{l}^{-}}$) in either pH, estimated in the absence or presence of the dendrimer inside the α-HL, was assessed using an alternative form of the Goldman-Hodgkin-Katz equation:

$$\frac{{P}_{{K}^{+}}}{{P}_{C{l}^{-}}}=\frac{{a}_{C{l}^{-}}^{trans}-{a}_{C{l}^{-}}^{cis}\cdot \exp (\frac{{{\rm{\Psi }}}_{rev}F}{R{T}_{m}})}{{a}_{{K}^{+}}^{trans}\cdot \exp (\frac{{{\rm{\Psi }}}_{rev}F}{R{T}_{m}})-{a}_{{K}^{+}}^{cis}}$$

where ${P}_{{K}^{+}}$ and ${P}_{C{l}^{-}}$ represent the permeabilities of the two ionic species, ${a}_{C{l}^{-}}^{trans},{a}_{{K}^{+}}^{trans},{a}_{C{l}^{-}}^{cis}\,{\rm{and}}\,{a}_{{K}^{+}}^{cis}$, denote the chemical activities of chloride and potassium ions on the *trans* and *cis* side of the membrane, respectively, *F* represents the Faraday constant, *R* is the ideal gas constant, and T_m_ is the absolute temperature (T_m_ = 295 K). The chemical activities were calculated according to Pitzer equation^90^, as the product of the activity coefficient,*γ*_*i*_ and the corresponding molar concentrations of the species *i*, *a*_*i*_ = *γ*_*i*_ *c*_*i*_ (a_KCl_ = 0.077 for c_KCl_ = 0.1 M, and a_KCl_ = 1.7 for c_KCl_ = 3 M).

## Electronic supplementary material

Supplementary information
