# Supplementary material for: Nanoscale Investigation of Generation 1 PAMAM Dendrimers Interaction with a Protein Nanopore
Source: Sci Rep. 2017 Jul 21;7:6167. doi: 10.1038/s41598-017-06435-1 (PMC5522495; doi:10.1038/s41598-017-06435-1)
Supplement: Supplementary file 1 — Supplementary information [file 41598_2017_6435_MOESM1_ESM.pdf]

Supplementary Information for:

Nanoscale Investigation of Generation 1 PAMAM Dendrimers Interaction with a Protein Nanopore

Alina Asandei[1#], Andrei Ciuca[2,#], Aurelia Apetrei[2], Irina Schiopu[1], Loredana Mereuta[2], Chang Ho Seo[3], Yoonkyung Park[4,] & Tudor Luchian[2,*]*

[1]Interdisciplinary Research Department, Alexandru I. Cuza University, Iasi, Romania.

[2] Department of Physics, Alexandru I. Cuza University, Iasi, Romania.

[3] Department of Bioinformatics, Kongju National University, Kongju, South Korea.

[4] Department of Department of Biomedical Science and Research Center for Proteinaceous Materials (RCPM), Chosun University, Gwangju, Korea.

[*]Corresponding authors (y_k_park@chosun.ac.kr and luchian@uaic.ro)

[#]These authors contributed equally

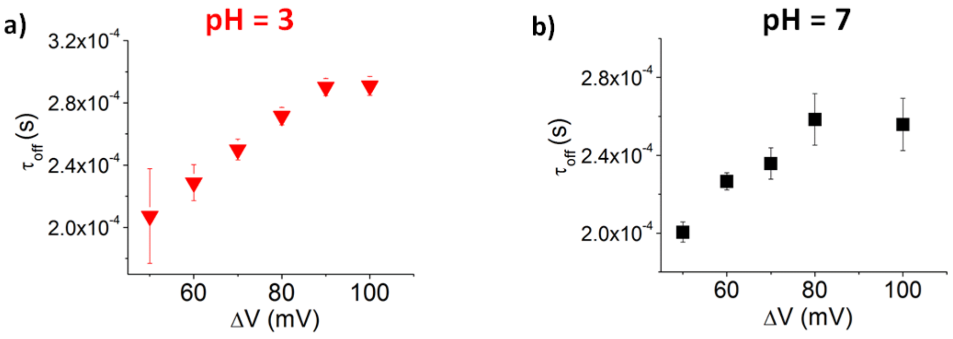

Fig. S1 Voltage-dependence of the durations (τ_{off}) of blockade events induced by PAMAM-G2 on the α-HL, at pH=3 ('▼' – 'down triangles', panel a) and pH=7 ('■' – 'black squares', panel b). Due to the differences in effective diameter (d) ($d_{PAMAM\ G2}$ ~ 2.3 nm and $d_{PAMAM\ G1}$ ~ 1.8 nm[1], the PAMAM-G2 dendrimer does not pass readily through the constriction region of the α-HL (d ~ 1.5 nm), and dissociates from the nanopore by returning to the *trans* side, against the electrophoretic force (see main text).

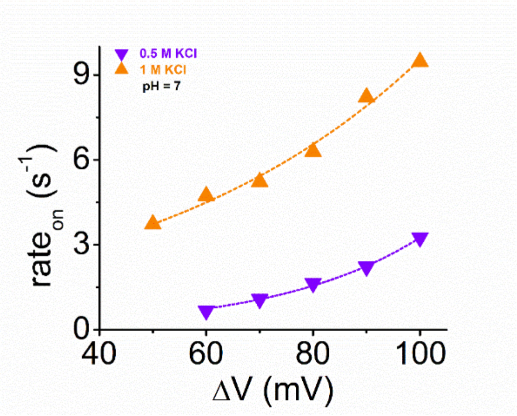

Fig. S2 Voltage-dependence of the PAMAM-G1 – α-HL association rates in electrolytic buffers containing 1 M KCl ('▲' – 'up triangles') and 0.5 M KCl ('▼' – 'down triangles'). The dotted lines represent the exponential fits of the data according to the Van't Hoff-Arrhenius law (see main text).

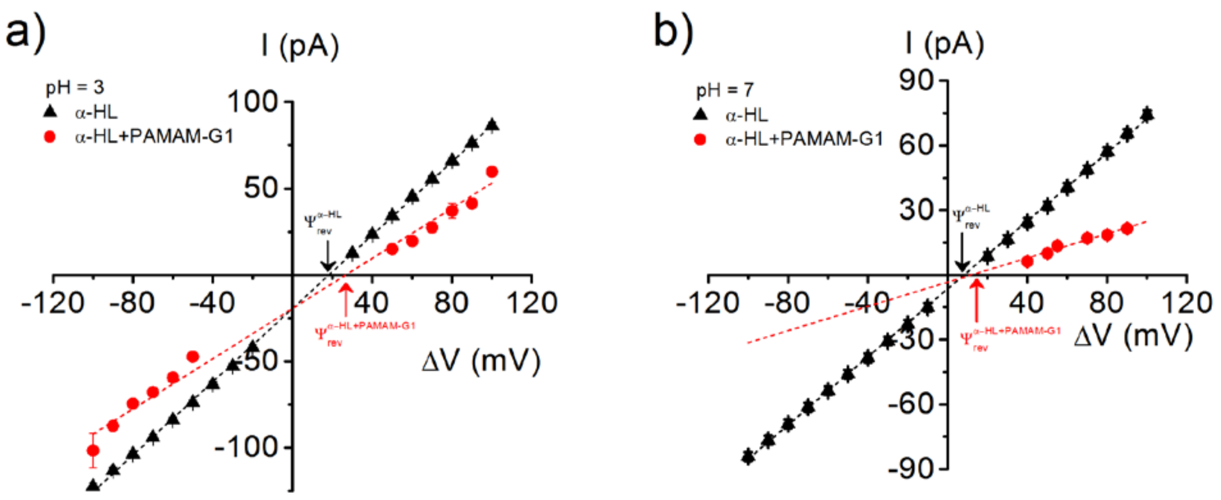

Fig. S3 I-ΔV diagrams for the ionic current measured across the α-HL nanopore in a salt gradient of 0.1M KCl (*cis*)/3M KCl (*trans*), in the absence (triangles) and transient presence of the PAMAM-G1 dendrimer inside the nanopore (circles), at pH=3 (a) and pH=7 (b). The reversal potential (Ψ_{rev}) estimated in either case ($\Psi_{rev;pH=3}^{\alpha-HL}$=18.32 mV, $\Psi_{rev;pH=3}^{\alpha-HL+PAMAM-G1}$=26.73 mV; $\Psi_{rev;pH=7}^{\alpha-HL}$=8.21 mV, $\Psi_{rev;pH=7}^{\alpha-HL+PAMAM-G1}$=11.84 mV), was later used to derive the ion selectivity of the dendrimer-free and dendrimer-blocked α-HL, at both pH's (see also *Materials and Methods*). Note the absence of data points for the case of dendrimer-blocked α-HL, at negative ΔV's and pH=7 (panel b). This is caused by the virtual absence of dendrimer inclusions inside the nanopore, as the electrophoretic force at negative ΔV's drives the dendrimer away from the nanopore. However, dendrimer-induced blockade events were still seen at negative ΔV's, but pH=3 (panel a); we propose that this is due to the contribution of electro-osmotic force acting on the dendrimer, which is *trans*-to-*cis* oriented at such negative potentials and, unlike the neutral pH case, much enhanced as a result of the increase in the anionic selectivity of the nanopore at pH=3.

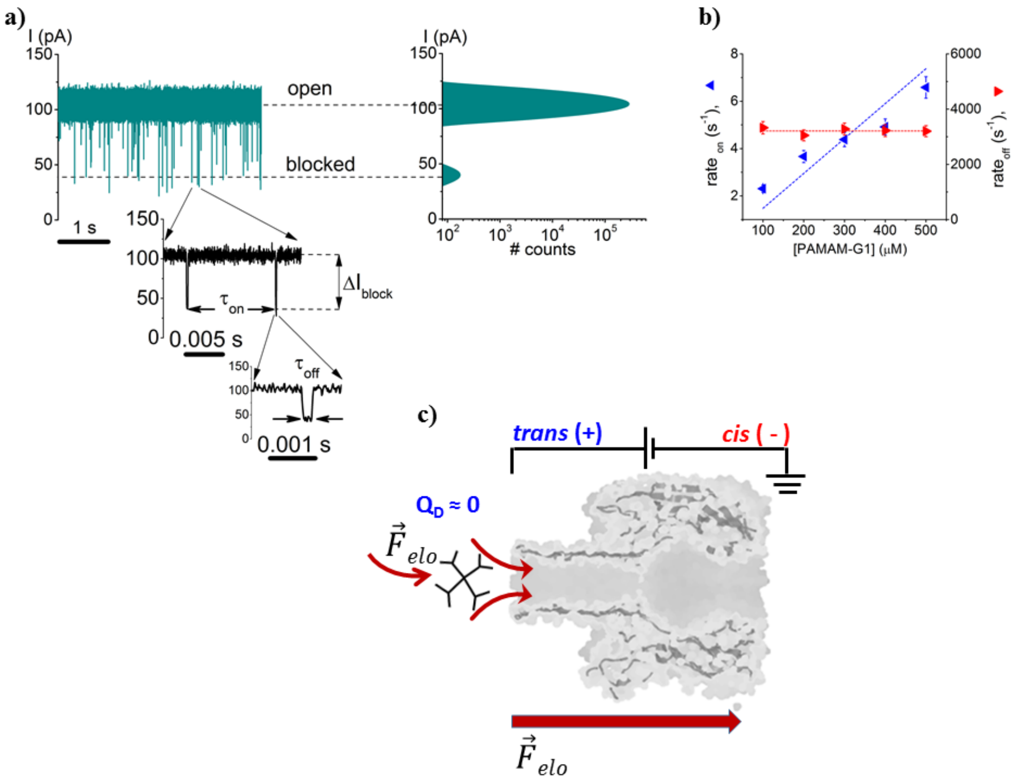

Fig. S4 The electro-osmotic-mediated PAMAM-G1 capture by the α-HL, at basic pH. In panel a we show a representative trace and all-events histogram of PAMAM-G1 – α-HL interactions, recorded at ΔV = +100 mV, in 1 M KCl, at basic pH (pH=10.3). The dendrimer was added on the *trans* side of the nanopore, at a bulk concentration of 500 μM. The zoomed-in traces in the inset show the main parameters used to describe the electrical signature of the PAMAM-G1 – α-HL interactions (*i.e.*, τ_{on}; inter-event time, τ_{off}; blockade duration and ΔI_{block}; current blockade amplitude). In panel b are shown the concentration-dependence of the dendrimer capture rate by the nanopore ($rate_{on}$; '◀' - 'left triangles'), and of the dissociation rates ($rate_{off}$; '▶' - 'right triangles'), respectively. From the linear fit of these data with constant ($rate_{on}$), and respectively zero slope functions ($rate_{off}$), the corresponding reaction constant rates were derived (k_{on} = 14.7 x $10^3 \pm 1.6$ x 10^3 s^{-1} M^{-1} and k_{off} = 3.2 x $10^3 \pm 47.8$ s^{-1}). Panel c shows a simplified view of the PAMAM-G1 capture inside the α-HL, at positive *trans*-applied voltage, mediated by the electro-

osmotic force ($\overrightarrow{F_{elo}}$, red arrow) which, due to the cation selectivity of the α-HL at pH=10.3, reverses its direction as compared to neutral and acidic pH's and guides the analyte towards the α-HL' β-barrel entry (see also main text). Note that at pH=10.3, the calculated bare charge on the dendrimer is null ($Q_D \approx 0$), and consequently the electrophoretic driving force $\overrightarrow{F_{elp}}$ exerted upon it equals 0.

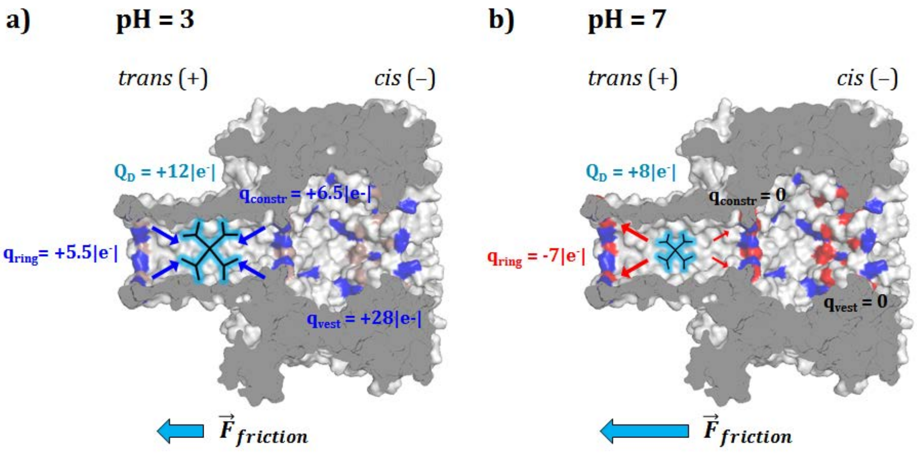

Fig. S5 Sketched view of the electrostatic interactions between the PAMAM-G1 dendrimer and the α-HL' nanocavity, at acidic and neutral pH. Basic amino acids lining the inner walls of α-HL are represented in blue, while acidic amino acids are represented in red. At pH=3 (panel a), the acidic residues are protonated (represented in faded pink), resulting in a net positive charge at the β-barrel opening (bare charge of q_{ring} = +5.5|e^-|), constriction (bare charge of $q_{constr.}$ = +6.5|e^-|) and in the vestibule domain (bare charge of $q_{vest.}$ = +28|e^-|). At pH=7 (panel b), the β-barrel opening is negatively charged (bare charge of q_{ring} = -7|e^-|), whereas the constriction and the vestibule regions are largely neutral ($q_{const.}$ = 0; q_{vest} = 0). At pH=3 (panel a), a transiting, positively charged dendrimer (bare charge of Q_D = +12|e^-|), interacts repulsively with the charged domains

inside the α-HL (corresponding forces are represented schematically in blue arrows), and we posit that this lowers the lumped friction force ($\vec{F}_{friction}$) experienced by the dendrimer inside the nanopore (see text). At pH=7 (panel b), the *trans*-to-*cis* moving dendrimer (calculated bare charge of $Q_D = +8|e^-|$), experiences electrostatic, attractive forces (schematically represented in red arrows) with the negatively charged β-barrel opening, resulting in a putative higher friction force ($\vec{F}_{friction}$), as compared to acidic pH.

Uni-dimensional formalism for the derivation of the dendrimer drift velocity inside the α-HL nanopore, under the collective influence of electro-osmotic and electrophoretic forces

In the model developed by Talaga and Li[2], and later corrected[3], is shown that the first-passage probability density function (p(t)) which describes the distribution of values of translocation times (τ_{off}) related to the sojourn of analytes inside a nanopore, in the presence of an electric field, is given by:

$$p(t) = \frac{L}{\sqrt{4\pi D t^3}} e^{-\frac{\left(L - v_{drift} t\right)^2}{4Dt}} \qquad (1)$$

where L represents the nanopore's length (L ~ 10 nm), D the diffusion coefficient of the analyte within the nanopore, and the drift velocity of the analyte (v_{drift}) accounts for both its interaction with the electric driving force and the electro-osmotic flow of water.

Neglecting the contributions from pressure gradients, the drift velocity of the dendrimer in the electric field along the α-HL is the vector sum of the electrophoretic and electro-osmotic

components. Because the α-HL is anion selective at acidic and neutral pH, the positively charged dendrimer moves *trans*-to-*cis*, opposite to the net flow of water carried by the *cis*-to-*trans* moving anions. That is, the electro-osmotic water flow opposes electrophoresis, and the drift velocity of a dendrimer moving along the electric field lines in the *trans*-to-*cis* direction within the nanopore, entering the formula (1), can be written as[4]:

$$v_{\text{drift}} = v_{\text{electrophoretic}} - v_{\text{electroosmotic}} = \mu \frac{\Delta V}{L} - \frac{1-\frac{P_{K^+}}{P_{Cl^-}}}{1+\frac{P_{K^+}}{P_{Cl^-}}} \frac{N_w I_{blocked}}{|e^-| S_{pore}[H_2O]} \qquad (2)$$

with S_{pore} as the average cross-sectional area of the α-HL (S_{pore} = 3.14 x 10^{-18} m^2), μ the electrophoretic mobility of the dendrimer within the nanopore, which can be expressed through its diffusion coefficient (D) as $\mu = \frac{z|e^-|D}{k_B T_m}$ (z and e^- are valence of the dendrimer and the elementary charge value, respectively, k_B is the Boltzmann constant, T_m the absolute temperature, equal to 295 K in our calculations), N_w represents the number of waters associated with each mobile ion (N_w ~10), [H_2O] the water concentration expressed as $\left(\frac{\text{number of molecules}}{\text{m}^3}\right)$ ([H_2O] ~ 3.35 x 10^{28} molecules/m^3), I_{blocked} is the net electric current transported while a dendrimer resides within the nanopore (see Table S2), P_{K+} and P_{Cl-} represent the permeabilities of potassium and chloride ion species through the nanopore when a single PAMAM-G1 dendrimer resides inside the α-HL, and ΔV is the applied transmembrane potential.

As anticipated, and due to its cationic charge, the presence of the dendrimer inside the nanopore alters the anionic charge selectivity of α-HL. Using the protocol described in the ***Materials and Methods*** section and experimental I-ΔV diagrams shown in Fig. S3, we arrived at

P_{K^+}/P_{Cl^-} ~ 0.703 at pH = 7 and P_{K^+}/P_{Cl^-} ~ 0.453 at pH = 3, for the free α-HL, and P_{K^+}/P_{Cl^-} ~ 0.601 at pH = 7 and P_{K^+}/P_{Cl^-} ~ 0.350 at pH = 3, for the dendrimer-blocked α-HL, respectively. The anionic charge selectivity of the dendrimer-blocked α-HL increases with ~ 17 % at pH = 7, and ~ 29 %, respectively, at pH = 3, in comparison to the dendrimer-free nanopore, and this is mainly due to the augmented cationic charge on the dendrimer at low as compared to neutral pH's (see Fig. 1).

As for the net charge ($Q = z|e^-|$) of the PAMAM-G1 dendrimer, we note that is stems from eight primary amines with pK_a ~ 9, four tertiary amines with pK_a ~ 5, and other two tertiary amines with pK_a ~ 2 [5]. We have estimated the bare net charge of the dendrimer as a function of pH, based on the Henderson-Hasselbalch equation: at pH = 7, Q = +7.96$|e^-|$, and at pH = 3, Q = +12.14$|e^-|$. We note that at the ionic strengths employed in our experiments (1 M KCl and 0.5 M KCl), the effective charge of the dendrimer is expectedly smaller than the calculated bare values, due to salt screening of the charged moieties on the analyte. However, an exact estimation of the reduction in the dendrimer's charge is not obvious, since the protonable amine moieties on the dendrimer - modelled as a spherical particle with cavities - cannot be viewed as uniformly distributed on the dendrimer's surface solely, rendering the quantitative evaluation of charge screening by counterions distribution within the Debye-Hückel formalism not trivial[6]. Thus, for qualitative, numerical estimations, we employed in the subsequent calculations the bare (un-screened) value for the net electric charge on the dendrimer (Q), with the specification that in this case, the resulting values for the diffusion coefficient are lower limits of the parameter.

By simple statistical analysis, the probability distribution (1) yields an expression for the average time (τ_{off}) spent by the dendrimer inside the α-HL:

$$\langle \tau_{\text{off}} \rangle = \frac{1}{\text{rate}_{\text{off}}} = \int_0^\infty t \frac{L}{\sqrt{4\pi D t^3}} e^{-\frac{\left(L - v_{drift} t\right)^2}{4Dt}} dt = \frac{L}{v_{drift}} \qquad (3)$$

By combining expressions 2 and 3, and the experimental data regarding (τ_{off}) values displayed in Fig. 4 (1 M KCl) and Fig. 6 (0.5 M KCl) (main text), we calculated the diffusion coefficient (D) of the dendrimer inside the nanopore at distinct applied values of the transmembrane potential (ΔV), under the experimental conditions employed (namely at pH = 7 and pH = 3 (1 M KCl), and distinct ionic strengths of the electrolytic solution, i.e. at 1 M and 0.5 M KCl, at pH=7 (see main text).

Table S1 Low limit, estimated values of the self-diffusion coefficient (D) of the PAMAM-G1, in all three experimental conditions employed, for different values of the applied transmembrane potential ΔV.

	pH = 3 (1 M KCl)	pH = 7 (1 M KCl)	pH = 7 (0.5 M KCl)
ΔV (mV)	D (cm^2s^{-1})	D (cm^2s^{-1})	D (cm^2s^{-1})
50	-	1.10E-8	-
60	2.33E-8	1.40E-8	-
70	2.64E-8	1.71E-8	0.89E-8
80	2.97E-8	1.92E-8	1.13E-8
90	3.43E-8	2.19E-8	1.21E-8
100	3.85E-8	2.35E-8	1.27E-8

Table S2 Experimentally measured, average values of the electric current through the α-HL, while a PAMAM-G1 dendrimer resides within the nanopore ($I_{blocked}$), for different values of the applied ΔV, electrolyte's pH and salt concentration

	pH = 3 (1 M KCl)	pH = 7 (1 M KCl)	pH = 7 (0.5 M KCl)
ΔV (mV)	$I_{blocked}$ (pA)	$I_{blocked}$ (pA)	$I_{blocked}$ (pA)
50	27	23.1	-
60	38.8	29.32	-
70	43.88	35.82	18.58
80	49.44	40.26	23.72
90	57.13	46.05	25.42
100	64.14	49.26	26.5

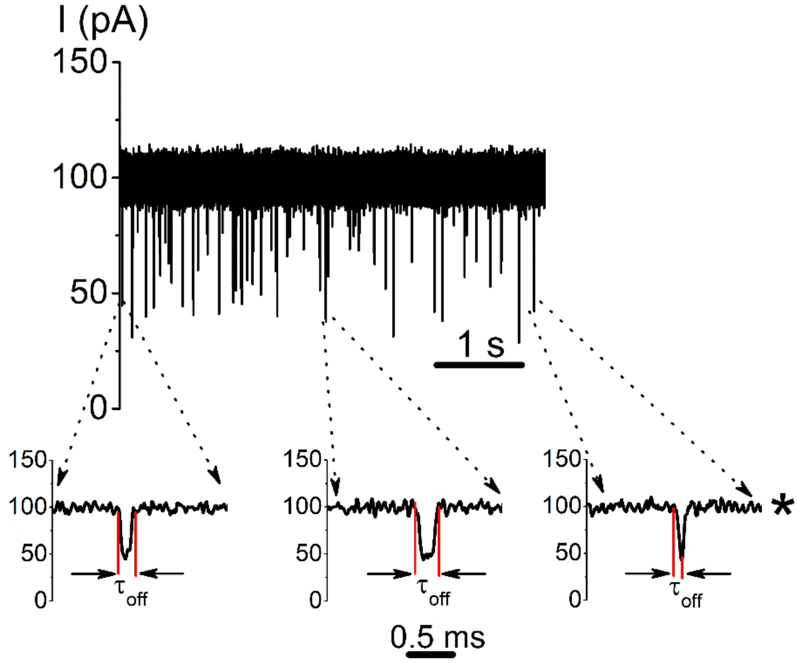

Fig. S6 The experimental setup-induced, low-pass filter effect, on the shape of dissociation events of the dendrimer from the nanopore. Selected traces showing stochastic blockades of the ionic current flow across the α-HL nanopore, by the interaction with the *trans*-added PAMAM-G1 dendrimer at ΔV = +100 mV, in 1 M KCl, at pH=7. Indicated below are zoomed-in traces excerpts, showing the shape of ionic current levels assigned to a transiently occupied nanopore by a single dendrimer. As it is apparent especially in the example marked by '*', the reduced bandwidth of the recording system (~ 10 kHz) filters out the recordings associated to such dendrimer dissociation events, so that the current signal no longer resembles a square-like shape. In such instances, and according to the reference presented in the main text, the blockade duration quantified by τ_{off}, was assessed by measuring the time elapsed from the start of the pulse to its first rising edge.

References

1. Maiti, P. K., Çağın, T., Wang G. & Goddard, W. A. Structure of PAMAM Dendrimers: Generations 1 through 11. *Macromolecules* **37**, 6236-6254 (2004).
2. Talaga, D. S. & Li, J. Single-Molecule Protein Unfolding in Solid State Nanopores. *J. Am. Chem. Soc.* **131**, 9287-9297 (2009).
3. Ling D. Y. & Ling, X. S. On the Distribution of DNA Translocation Times in Solid-State Nanopores: an Analysis Using Schrodinger's First-Passage-Time Theory. *J. Phys. Condens. Matter* **25**, 375102 (2013).
4. Mereuta, L. *et. al.* Slowing Down Single-Molecule Trafficking through a Protein Nanopore Reveals Intermediates for Peptide Translocation. *Sci. Rep.* **4**, 3885 (2014).
5. Castagnola, M. *et. al.* Characterization of Dendrimer Properties by Capillary Electrophoresis and Their Use as Pseudostationary Phases. *Electrophoresis* **23**, 1769-1778 (2002).
6. Maiti, P. K. & Messina, R. Counterion Distribution and ζ-Potential in PAMAM Dendrimer. *Macromolecules* **41**, 5002-5006 (2008).
